# Infection rate and genetic diversity of *Giardia duodenalis* assemblage C in Iranian stray dogs, targeting the *glutamate dehydrogenase* gene

**DOI:** 10.14202/vetworld.2021.419-425

**Published:** 2021-02-16

**Authors:** Asghar Fazaeli, Mohammad Hasan Kohansal, Adel Spotin, Ali Haniloo, Abbasali Nourian, Alireza Khiabani, Abolghasem Siyadatpanah, Roghayeh Norouzi, Veeranoot Nissapatorn

**Affiliations:** 1 Department of Parasitology and Mycology, School of Medicine, Zanjan University of Medical Sciences, Zanjan, Iran; 2 School of Medicine, Bam University of Medical Sciences, Bam, Iran; 3 Immunology Research Center, Tabriz University of Medical Sciences, Tabriz, Iran; 4 Student Research Committee, Tabriz University of Medical Sciences, Tabriz, Iran; 5 Ferdows School of Paramedical and Health, Birjand University of Medical Sciences, Birjand, Iran; 6 Department of Pathobiology, Faculty of Veterinary Medicine, University of Tabriz, Tabriz, Iran; 7 School of Allied Health Sciences, Research Excellence Center for Innovation and Health Products, Walailak University, Nakhon Si Thammarat, Thailand

**Keywords:** genetic variation, *Giardia lamblia* assemblage C, *glutamate dehydrogenase*, Iran, stray dogs

## Abstract

**Background and Aim:**

*Giardia duodenalis* is one of the most common enteric protozoan parasites in vertebrates, such as humans, domestic and wild animals, causing giardiasis. To the best of our knowledge, little is known about the genetic diversity of *G. duodenalis* assemblages. This study aimed to identify genetic diversity of *G. duodenalis* assemblages in Iranian stray dogs.

**Materials and Methods:**

A total of 450 fecal samples were collected from 2015 to 2016 from stray dogs of Northwest Iran. All specimens were observed microscopically following concentration and flotation techniques. Subsequently, DNA samples were extracted, amplified, and sequenced targeting the *glutamate dehydrogenase* gene.

**Results:**

The overall prevalence of *G. duodenalis* in infected dogs was estimated at 1.6%, based on microscopic and molecular diagnoses. Sequencing and phylogenetic analyses indicated a high level of genetic diversity of assemblage C (haplotype diversity; 0.802).

**Conclusion:**

The pairwise sequence distances between the identified isolates of assemblage C showed an intradiversity of 0.3%-1.3% and identity of 98.7%-100%. Current findings indicate that a significant genetic diversity of *G. duodenalis* assemblage C haplotypes is unequivocally circulates among stray dogs in Northwest Iran.

## Introduction

The relationship between humans and dogs was discovered historically 15,000 years ago [1]. Despite the benefits of dogs to humans, their role in the transmission of important zoonotic diseases should not be underestimated [2]. It estimates that dogs can carry more than 60 zoonotic infections that can pose potential public health problems. *Giardia duodenalis* (syn. *Giardia intestinalis* and *Giardia lamblia*) is one of the zoonotic protozoan flagellate unicellular parasites that infect a wide range of vertebrates, such as humans and dogs. Giardiasis occurs with ingestion of the cysts, either through contaminated water, food, and materials or through host-to-host contact [3,4]. Genotypes of *G. duodenalis* from different host species are morphologically indistinguishable. However, some molecular methods using semi-conserved genes include *glutamate dehydrogenas*e *(gdh)* [5], *small subunit ribosomal locu*s *(18S rRN*A) [6], *elongation factor-1 (ef-1*) [7], and *triose phosphate isomerase (tpi)* [8] can be useful in discriminating genotypes/assemblages [6,9]. Based on genetic analysis and host specificity, eight distinct assemblages of *G. duodenalis* (A-H) have been reported [7].

Assemblages A and B isolates are potentially zoonotic and have been reported in a broad range of hosts, including humans, dogs, cats, livestock, and wildlife [10,11]. In contrast, C and D, E, F, G, and H of the assemblage most commonly infect canines [12,13], livestock, the same as cattle, pig and sheep [14], cats [15], rats [16], and marine mammals [17]. Dogs are predominantly infected with host-adapted assemblages C and D of *G. duodenalis;* however, potentially zoonotic assemblages found in humans (assemblages A and B) may have also been isolated from dogs [18,19].

In terms of a public health perspective, it is imperative to distinguish specific host assemblages (assemblages A and B) from *G. duodenalis* from those that have zoonotic potential, using a molecular approach, such as PCR-based procedures [20].

According to the previous studies, it demonstrated the prevalence of different infections with zoonotic genotypes in different geographical areas; 5.5% in Australia, 61% in Thailand, and 80.5% in Belgium [21]. There are no known reasons to support the different rates of infection and it deserves further studies. In Iran, several studies have been conducted in an epidemiological survey of zoonotic canine intestinal parasites [22,23]. There is, however, a few data on the distribution of *G. duodenalis* assemblages in the canine community, particularly in Zanjan of Northwest Iran.

This phylomolecular study was aimed to determine the infection rate and heterogeneity features of *G. duodenalis* assemblages isolated from infected stray dogs to integrate the baseline data on canine genetic diversity in this part of Iran.

## Materials and Methods

### Ethical approval

The study proposal was reviewed and approved by the Institutional Research Ethics Committee of Zanjan University of Medical Sciences (Reference number A-12-153-8).

### Study area and period

The study was conducted on stray dogs in the city of Zanjan (latitude: 36°40′24″ N; longitude: 48°28′43″ E), which is located in Northwest Iran. Agriculture and animal husbandry are the most important occupations of the inhabitants of this province. There were 2,366,411 farm animals in the province [22]. The study was conducted from January 2015 to December 2016.

### Sample collection

A total of 450 fecal samples from stray dogs were collected from selected areas of Zanjan Province, such as streets and farms in rural areas of different parts of Zanjan city. Fecal samples were placed in labeled sterile “Ziploc” bags and immediately transported to the research center of the Zanjan University of Medical Sciences, on the same day of sample collection. All samples were examined by microscopy and later analyzed by the polymerase chain reaction (PCR) targeting the *gdh* gene. *G. duodenalis* cysts were isolated from fecal samples using a sucrose gradient centrifugation technique [24]. The presence of the parasite was identified under a light microscope (Olympus BX41TF, Tokyo, Japan) examination with 400× and the morphological identification was confirmed by the size, shape, and internal structures compatible with the standard reference.

### DNA extraction and PCR

DNA was extracted from 200 mg of each fecal sample using the AccuPrep Stool DNA extraction kit (Bioneer Corporation, Soul, South Korea). The manufacturer’s protocol was used with the following modifications: 600 μL of digestion buffer (100 mM NaCl, 10 mM Tris-HCl pH 8.0, and 25 mM EDTA) was mixed with samples. Glass pearls (0.45-0.52 mM in diameter) were added to the mixtures, and the samples were vortexed for 10 min. Subsequently, these samples were subjected to seven cycles of freeze/thawing using liquid nitrogen and boiling water to disrupt the cyst walls. At this stage, 20 μL of proteinase K (final concentration of 200 μg/mL) and 40 μL of 2% sodium dodecyl sulfate were added to each sample and placed in a water bath at 60°C overnight. Finally, the parasite DNA was extracted according to the protocol recommended by the manufacturer. The concentration of extracted DNAs was measured by NanoDrop (Thermo Scientific 2000C, Wilmington, USA). The extracted DNAs were stored at −20°C until analysis. A DNA fragment (432 bp) from the *gdh* gene was amplified with primers reported by Read *et al*. [25]. The amplification reactions were provided in 25 μL of volume, containing 12.5 μL Master Mix (Ampliqon, Odense, Denmark, 1 μL of primers 10 pmoL), 1 μL of templates DNA, and 10.5 μL of deionized distilled water. PCR was carried out with the following amplification conditions: 1 cycle of 5 min primary denaturation at 95°C, followed by 40 cycles of denaturation at 94°C for 30 s, annealing at 50°C for 30 s, and extension at 72°C for 60 s, ending with a final extension at 72°C for 7 min. Finally, the products of the second PCR were visualized by electrophoresis on 1% agarose gel stained with Safe Stain (SinaClon, Tehran, Iran).

### DNA sequencing and phylogenetic analysis

Amplicons from positive fecal samples were sequenced (Munpyeongseo-ro, Daedeok-gu, Daejeon, South Korea) targeting the *gdh* gene in both directions using the GDHeF/GDHiF primers. The ambiguity sites of sequences were edited according to the reference sequence (RefSeq) using the Sequencher Tm v.4.1.4 software based on IUPAC codes (Gene Codes Corp., Michigan, USA). To assess the genetic diversity, the DnaSP software was used according to the analysis of molecular variance (AMOVA) to calculate the diversity of haplotypes (Hd); nucleotide diversity (π); and number of haplotypes (Hn). A sequence distance matrix pairwise was computed using the DNASTAR MegAlign program to show the percent identity (%) and intraspecies diversity of *G. duodenalis* assemblages between geographical sequences of the *gdh* gene. To authenticate the genetic relationships between identified *G. duodenalis* assemblages, a phylogenetic tree was generated by the MEGA 5.05 software (Pennsylvania State University, www.megasoftware.net) based on the maximum likelihood algorithm and the Kimura 2-parameter model. The distance scale was estimated at 0.02. *G. ardeae* (Accession no; AF069060) was addressed as an outgroup branch. Multiple alignments between the amino acid and nucleotide sequences of *G. duodenalis* were performed based on Clustal W method (BioEdit software, version 7.0.5, Ibis Therapeutics, California, USA).

## Results

### Parasitological diagnostic, PCR, sequencing of *gdh*, diversity indices, and pairwise sequence distance matrix

**Figure-1:**
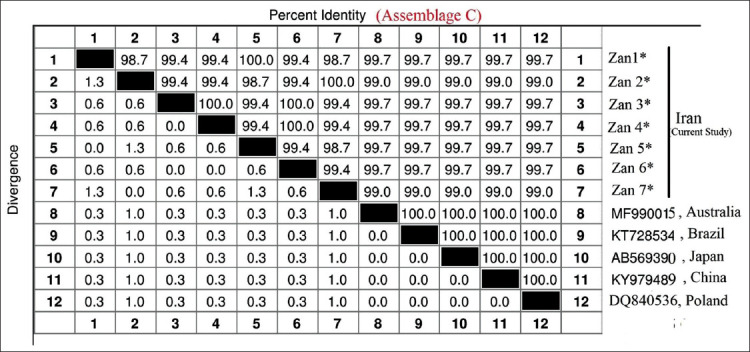
The sequence pairwise distances (divergence and percentage of identity) of isolates from *Giardia duodenalis* assemblage C identified (Zan1* to Zan7*) between the sequences circulating in the GenBank database determined by the *gdh* gene.

Microscopic examination and PCR amplification (432 bp fragment) of the *gdh* gene were identified in 7 (1.6%) samples consistent with *G. duodenalis*. Sequence analysis revealed that seven positive samples belonged to assemblage C. In the consensus position of 314 bp in the assemblage, 12 variable (polymorphic) sites were also detected (Table-1). The numbers of haplotypes (Hn), the number of isolates, diversity indices, and neutrality indices of isolates from *G. duodenalis* assemblage C are shown in Table-1. Sequencing and phylogenetic analyses indicated a high level of genetic diversity of *Giardia* assemblage C containing seven new haplotypes (Hd; 0.802) (Table-1). The pairwise sequence distances between the identified isolates of *Giardia* assemblage C showed an intraspecies diversity of 0.3%-1.3% and identity of 98.7%-100% (Figure-1).

**Table-1:** Diversity indices of *Giardia duodenalis,* assemblage C isolated from stray dogs and based on *glutamate dehydrogenase* sequences.

Parasite	Diversity indices				

	n	Hn	Hd±SD	Nd (π)	No. of polymorphic sites
*Giardia duodenalis assemblage C*	7	7	0.802±0.094	0.00895	12

N=Number of isolates, Hn=Number of haplotypes, Hd=Haplotype (gene) diversity, Nd=Nucleotide diversity.

### Multiple sequence alignment and phylogenetic tree

**Figure-2:**
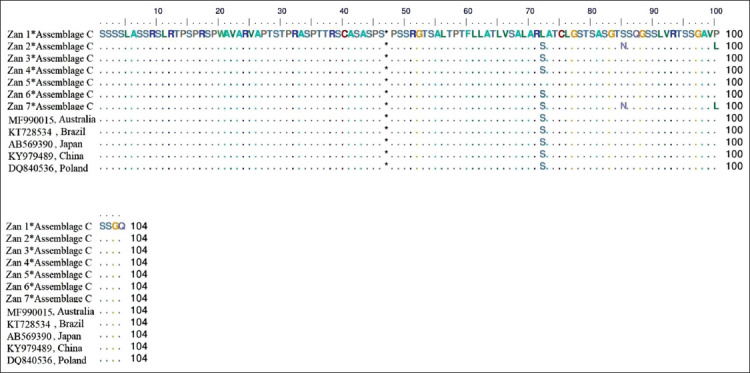
Amino acid sequence alignment of *gdh* gene based on identified haplotypes of *Giardia duodenalis* assemblage C (Zan1* to Zan7*).

**Figure-3:**
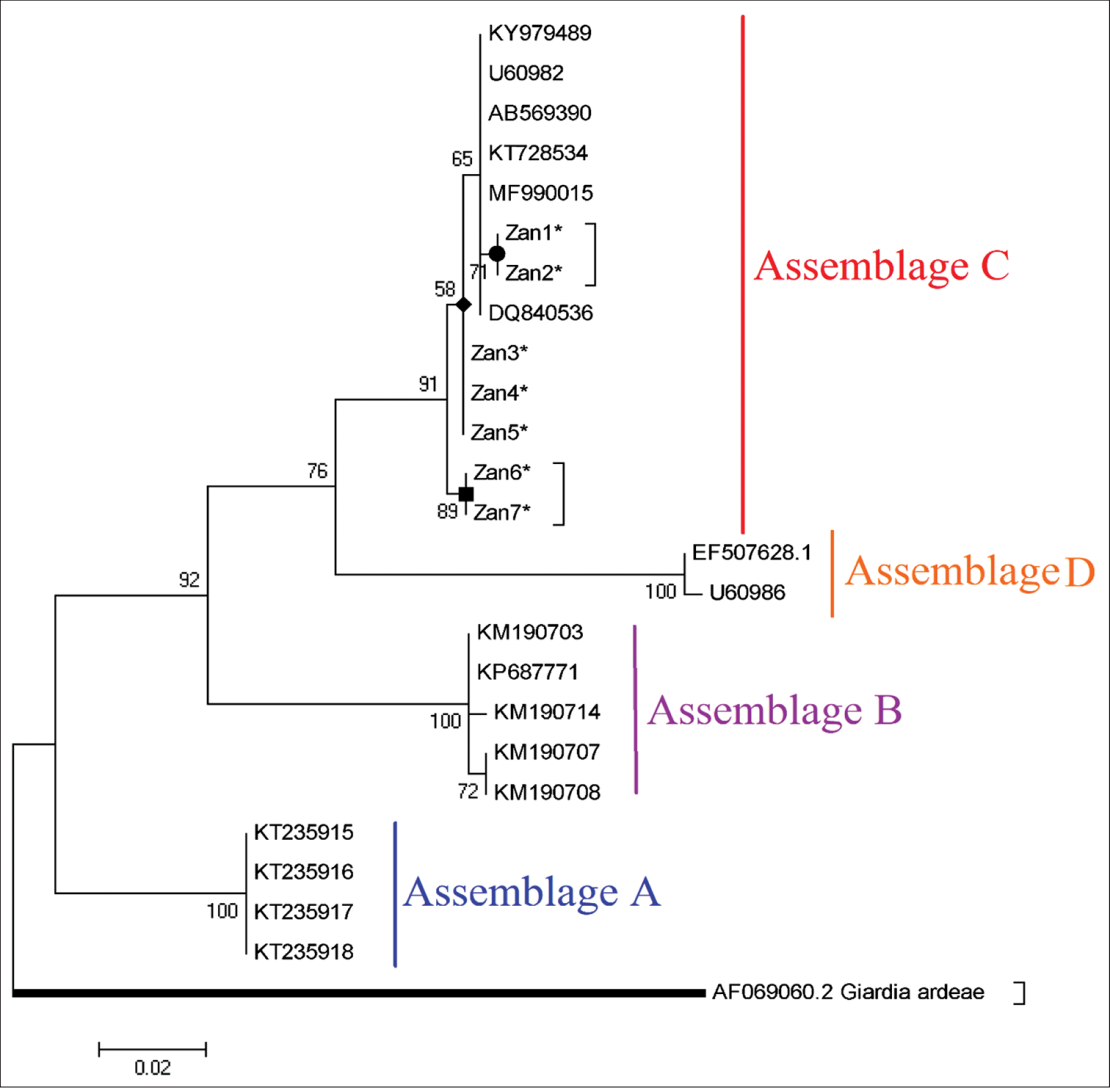
Phylogenetic analysis of canine isolates of *Giardia duodenalis* using the *gdh* gene based on the maximum likelihood algorithm with a Kimura 2-parameter model. The distance scale was estimated at 0.02. *G. ardeae* (Accession no; AF069060) was addressed as an outgroup branch. The relevant sequences for this study are marked with an asterisk (*). Bootstrap values (% based on 1000 replicates) of more than 50% are indicated.

In our targeted regions of sequences, no insertion or deletion (*I*ndel) mutations were found in *G. duodenalis* assemblage C. In comparison to globally RefSeqs, the multiple amino acid alignments of *G. duodenalis* assemblage C (Zan1*-Zan7*) indicated the occurrence of two non-synonymous substitutions in codons 72, 85, and 100, where Seine (S) replaced a leucine (L), asparagine (N) replaced a S, and L replaced a proline (P), respectively (Figure-2). To authenticate the taxonomic status of sequenced isolates, a maximum likelihood phylogenetic tree was constructed and inferred from *gdh* sequences. The topology of the identified positive isolates showed that *G. duodenalis* assemblage C (Zan1*-Zan7*) is placed in its specific clade adjacent to assemblage D clade (Figure-3).

## Discussion

The zoonotic transmission of *G. duodenalis* from canine to human is an important public health issue that requires further investigation [10,26,27]. Previous molecular epidemiological studies have reported potentially zoonotic assemblages A and B in the canine population [28,29]. Therefore, sufficient explanation has been provided to demonstrate the risk of transmission to humans worldwide [30-33].

On the other hand, it should be noted that the preferred option to conclusively determine the zoonotic transmission of *G. duodenalis* genotypes from canines to humans is those evaluated genotype/subtype at multiple loci of all positive pet (canine) samples to their humans in the same spatial and temporal settings [21].

The current study was conducted to determine the genotype diversity of *G. duodenalis* isolates from stray dogs in the city of Zanjan, northwest of Iran. In our study, *G. duodenalis* was detected 1.6% of fecal samples with microscopic examination and PCR method. Other studies in Iran have reported a low prevalence of *G. duodenalis* infections using microscopic examination, such as 0.07% of the prevalence found in 98 stray dogs and 0.09% of the infection rate with *G. duodenalis* of 100 samples studied [34,35]. Our findings are practically identical to a study previously reported in Taiwan, which showed that 0.09% of stray dogs were infected with *G. duodenalis* using PCR analysis [36]. However, the present result shows contrarily to those reported a high prevalence of *G. duodenalis* infection in the canine population. Silva *et al*. [37] showed that 28% of 100 canine fecal samples were positive for *G. duodenalis* infection [37], and later, Qi *et al*. [18] also reported a high infection rate (17.3%) of *Giardia* found in 359 samples [18]. Taken into consideration, these data suggest that there are various factors affecting the prevalence of *G. duodenalis* infection, such as canine population (indoor, stray, and shelter) and the use of diagnostic methods (microscopy, ELISA, or PCR) [38].

From this study, the detection method was considered the main factor affecting the prevalence rates [39], although PCR is more sensitive method to detect low numbers of *G. duodenalis* in dogs’ feces compared to microscopy [40]. Surprisingly, our study showed similar results between these two methods used to detect the presence of *Giardia* in our canine communities. However, recent molecular tools, such as a PCR-based diagnostic system, and sequencing with housekeeping genes, such as *18S rRNA, bg*, *gdh*, *ef-1*, and *tpi*, are the most frequently used loci for the differentiation of *Giardia* at genotype levels [10,41].

Since the *gdh* loci has been used to differentiate the common assemblages of *G. duodenalis* and genetic diversity, the *gdh* locus proved to be stable for the isolates identified in different host species and geographic locations [5,11,16,42,43]. In the current study, the molecular analysis of seven isolates of *G. duodenalis* showed the presence of C genotype. It is noteworthy to mention that all genotypes were canine adapted and were not found isolates from other zoonotic assemblages. Our results are in line with Abe *et al*. [44] study that used a direct sequencing of *gdh* loci that showed canine-specific genotypes, assemblage D [44]. The preliminary investigation of dogs in Hungary has shown that no zoonotic potential of *Giardia* genotypes and assemblages D and C in dog-specific strains of *G. intestinalis* has been reported [45]. Ouza *et al*. [15] conducted a study in 19 dogs using *gdh* coding genes and showed only the host-adapted assemblages C and D (7 and 20, respectively) [15]. Zhang *et al*. [46] in a study of 159 fecal samples from stray dogs using *bg*, *gdh*, and *tpi* genes demonstrated that genetic assemblages C and D of *G. duodenalis* were present among dogs in China [46]. Based on the above findings, they suggest that the result of the genotype strongly depends on the target sequence examined. Furthermore, the origin of the dogs’ fecal samples can affect the genotypic outcome. The present study was conducted on fecal samples from stray dogs, which is most likely that these animals are decisively exposed to each other and the transmission of dog-specific genotypes is favored by the close contact between a large number of dogs living together [45,47].

In contrast to our results, other studies with one or multiloci, such as *18SrDNA*, *bg*, and *tpi*, reported different zoonotic assemblages. A study in China conducted by Zheng *et al*. [6] demonstrated that most of the dogs were infected with assemblage A, which is potentially zoonotic potential [6]. In a study conducted in Japan on 24 dog feces, showed that 14 of the 24 sequenced samples belonged to assemblage A and 3 of them had both sequences of assemblages A and D (A/D) [29]. Adell-Aledon *et al*. [28] also showed the potential for transmission of zoonotic assemblages in Spain [28]. Results of their studies demonstrated zoonotic assemblages A and B, host-specific assemblages C and D. Also using genotyping based on multilocus sequences of the genes *gdh* and β*-giardin*, a number of inter-assemblage mixed infections, including A+B, A+D, and A+B+D, have also been reported. Genotyping and further subtyping have relied primarily on the sequence analysis on fragments of single-copy gene targets, such as *tpi, b-giardin* (*bg*), or *gdh*. However, this approach is now being reexamined because mixed infections may not always be distinguished by analyzing a single-locus and conflicting classification between loci [48]. This finding suggests that zoonotic or zooanthroponotic transmission rarely or infrequently occurs with respect to the humans and animals studied. To the best of our knowledge, this is the first molecular study on *Giardia* genotyping of stray dogs in this area. Although no zoonotic assemblages have been reported in the present research, study in canine and human populations using genotyping based on multilocus sequences and other valid molecular tools to obtain comprehensive information about *G. intestinalis* is strongly recommended.

## Conclusion

Our finding indicates, for the first time, that the significant genetic diversity of *G. duodenalis* assemblage C haplotypes is unequivocally circulating among stray dogs in Northwest Iran. This is therefore highly recommended for future studies with a larger sample size conducted in different parts of Iran to identify the genetic diversity of *G. duodenalis* in stray dogs and the association with human pathogenic strains of this protozoan parasite.

## Authors’ Contributions

AF, AN, and AH conceived and designed the experiments. AS, AF, and MHK contributed to the analysis and interpretation of data. MHK wrote the manuscript, while AK, ASi, VN, and RN assisted in writing and revision of the manuscript. All authors have read and approved the final manuscript.
